# A Rare Type of Primary Internal Hernia Causing Small Intestinal Obstruction

**DOI:** 10.1155/2016/3540794

**Published:** 2016-11-23

**Authors:** Sibabrata Kar, Vandana Mohapatra, Pratap Kumar Rath

**Affiliations:** ^1^Department of General Surgery, Shri Ramachandra Bhanj (S.C.B.) Medical College, Cuttack, Odisha 753007, India; ^2^Department of Obstetrics & Gynaecology, All India Institute of Medical Sciences (AIIMS), Bhubaneswar, Odisha 751019, India

## Abstract

Primary internal hernias are extremely rare in adults. They are an important cause of small intestinal obstruction and lead to high morbidity and mortality if left untreated. Clinical presentation of internal hernia is nonspecific. Imaging has been of limited utility in cases of acute intestinal obstruction; moreover, interpretation of imaging features is operator dependant. Thus, internal hernias are usually detected at laparotomy and preoperative diagnosis in an emergency setting is either difficult or most of the time not suspected. We report herein a case of a 45-year-old male who presented with acute intestinal obstruction which was attributed later to a very rare type of internal hernia on exploratory laparotomy. A loop of ileum was found to enter the retroperitoneum through a hernia gate which was located lateral to the sigmoid colon in the left paracolic gutter. The segment of intestine was reduced and the hernial defect was closed. Our finding represents an extremely rare variant of retroperitoneal hernias.

## 1. Introduction

An internal hernia (IH) is a protrusion of intestines or other abdominal organs through a normal or abnormal orifice in the peritoneum or mesentery, occasionally leading to strangulation or incarceration. Internal hernias (IH) are a rare cause of acute abdomen and intestinal obstruction in adults. IH has a reported autopsy incidence of 0.2 to 0.9% and is the cause of small bowel obstruction in 0.6 to 5.8% of the cases [1]. However, if strangulated and left untreated, internal hernias have an overall mortality greater than 50% [2]. Preoperative suspicion and diagnosis in an emergency setting are difficult due to rarity of the entity, nonspecific clinical presentation, and limited utility of imaging in cases of acute intestinal obstruction [3]. We describe here a case of a rare type of primary internal hernia presenting as acute intestinal obstruction in a 45-year-old Indian male.

## 2. Case Presentation

A 45-year-old male was admitted to the emergency department with complaints of acute onset severe abdominal pain and vomiting for three days. He was earlier evaluated and treated at a local hospital where he was kept on conservative management with insertion of nasogastric tube for 24 hours. But his symptoms were further aggravated with increased bouts of vomiting; as a result he was referred to our institute for further management. The patient had no history of similar attack in the past. He did not report any fever, dysuria, and change in bowel habit or hematochezia. There was no history of any comorbid illness, past surgical intervention, or trauma. On general examination, pulse rate was 98/minute and blood pressure was 130/80 mm of Hg. Patient was afebrile and tachypneic. Abdominal distension and tenderness were detected on abdominal examination. Bowel sounds were decreased. Digital rectal examination was normal. Rest systemic examination revealed no abnormality. Abdominal radiography in the upright position showed dilated small intestinal loops with multiple air-fluid levels suggesting intestinal obstruction. Ultrasonography of the abdomen revealed gaseous distension of the bowel loops. Routine blood investigations were within normal limit.

**Figure 1:**
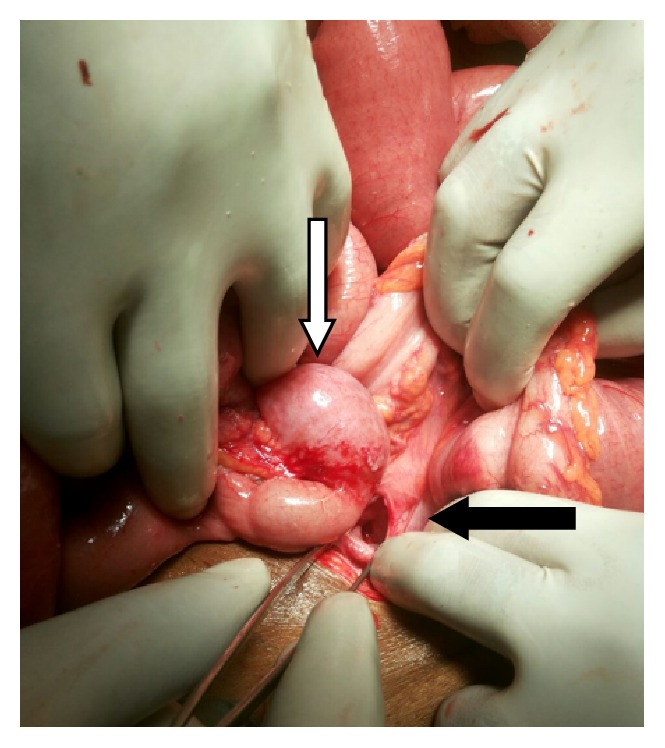
The hernia defect located lateral to sigmoid colon in the left paracolic gutter (black arrow); the bowel loop after reduction (white arrow).

**Figure 2:**
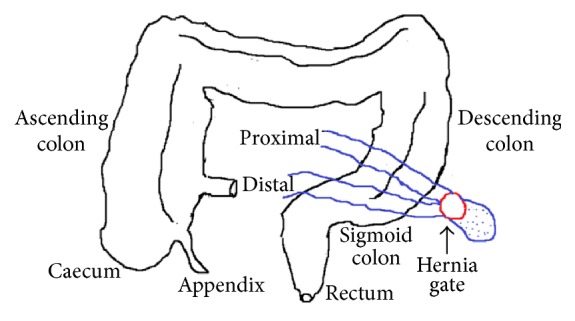
Schematic representation of intraoperative findings.

**Figure 3:**
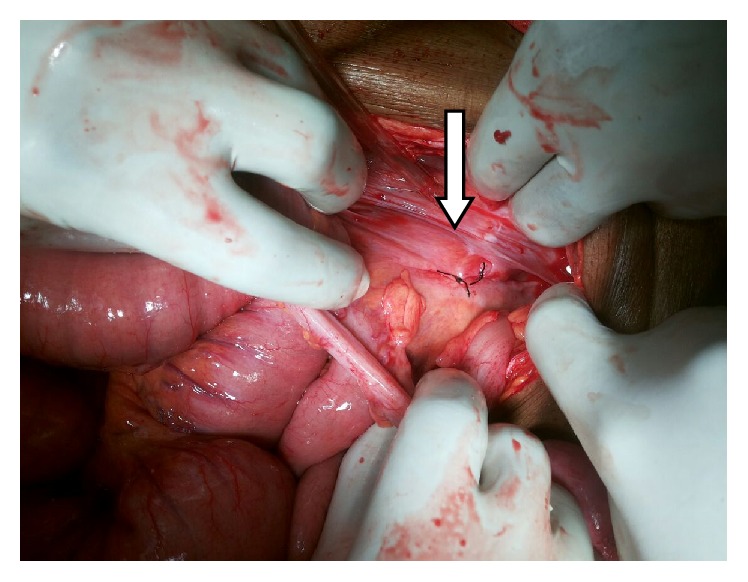
The repaired hernia defect after primary closure with nonabsorbable suture. White arrow refers to the repaired primary hernia defect.

The patient was planned for exploratory laparotomy in view of acute intestinal obstruction. On laparotomy, a loop of ileum was found to enter the retroperitoneum through a hernia gate (defect of 2.5 cm) located lateral to the sigmoid colon in the left paracolic gutter (Figures 1 and 2). The small intestinal loop was incarcerated at the neck of the hernia sac which was reduced after widening the hernia gate. The segment of intestine was found to be viable after reduction for which resection was not required. The hernia gate was closed with nonabsorbable interrupted suturing (Figure 3). There was no malrotation of the gut. Postoperative recovery was uneventful and the patient was discharged on the sixth postoperative day. He is now on follow-up and is doing well for the past six months after surgery without any symptoms.

## 3. Discussion

Internal hernias are either congenital or acquired, the latter constituting the majority. Important causes of acquired internal herniation in adults are previous abdominal surgery (mainly liver transplantation and bariatric procedures like gastric bypass), trauma, peritoneal inflammation, or ischemic changes [4]. Primary or congenital internal hernias in adults are extremely rare. Congenital internal abdominal hernias (CIAH) are either retroperitoneal or formed from congenital anomalous openings lacking a true peritoneal sac. Retroperitoneal hernias are further classified by Ghahremani into paraduodenal (30–53% of CIAH), foramen of Winslow (6%–10%), pericecal (10%–15%), intersigmoid (4%–8%), pelvic, and paravesical hernias (6%), whereas hernias formed from congenital anomalous openings can be categorized as transmesenteric (8%–10%), broad ligament (4–7%), or transomental hernias (1–4%) [5]. In our case, a retroperitoneal hernia was located in the left paracolic gutter lateral to the sigmoid colon. Thorough MEDLINE search revealed only few case reports of IH with the gate located in the paracolic gutter adjacent to the colon [5, 6]. These types have not been included in the current classification. Our finding contributes to the current literature.

Patients may be asymptomatic or present with clinical symptoms of small bowel obstruction (SBO) as the most frequently herniated organ is the small bowel. Acute abdomen is seen with ischemia and late cases of perforation [7]. Symptom severity relates to duration and reducibility of the hernia and the presence or absence of incarceration and strangulation [8]. Symptoms of intestinal obstruction in CIAH in adults are similar to symptoms due to other causes of SBO with acute onset of abdominal pain, tenderness, nausea, vomiting, and abnormal bowel sounds. Clinical presentation is nonspecific posing a diagnostic challenge preoperatively. This eventually can lead to diagnostic delays and the resultant increase in rates of ischemia, gangrene, and bowel resection.

Computed tomography (CT) now plays an important role in the evaluation of intestinal obstruction and acute abdomen [1]. Both radiographs and ultrasonography (23% detection) are poor in detecting the etiology of intestinal obstruction. Multidetector CT can identify the specific site and severity of obstruction (partial versus complete), closed loop, and multiple segments of obstruction [9]. It determines the etiology as well by identifying internal hernias and extraluminal lesions, such as masses, adenopathy, soft tissue infiltration, and vascular anomalies. CT features of IH include observation of a saclike mass or cluster of dilated small bowel loops at an abnormal anatomic location in the presence of SBO and observation of an engorged, stretched, or displaced mesenteric vascular pedicle and of converging vessels at the hernia orifice [10]. Complications such as ischemia, necrosis, or perforation and inflammatory changes can also be established on multidetector CT [11]. CT scan is thus a valuable tool in the early diagnosis and planning of surgical exploration in patients with IH and radiologists need to be familiar with the characteristic CT findings. However, the CT diagnosis of IH remains difficult even after its disseminated accessibility and use [9]. Review of cases showed that IH was often diagnosed at exploratory laparotomy performed for intestinal obstruction.

Timely surgical intervention based on clinical suspicion and/or CT scan findings is warranted for the management of IH presenting with intestinal obstruction [2]. Reduction of the strangulated intestinal segment should be done as early as possible to prevent intestinal ischemia, necrosis, and perforation and thereby reduce resection rates [12]. Hernia defects should be closed with nonabsorbable sutures in order to prevent recurrence of internal herniation through the same orifices in the future. Recently laparoscopic technique has also been found to be useful for diagnosis and treatment of intestinal obstruction [1, 2].

## 4. Conclusion

Internal hernias are a rare but important cause of intestinal obstruction given the high mortality associated, nevertheless still often underdiagnosed. Primary internal hernias should be kept in the differential diagnosis of acute intestinal obstruction in adults with no previous history of surgery or trauma. Since physical examination findings are nonspecific, a high index of clinical suspicion along with urgent CT is suggested to aid in the preoperative diagnosis of IH. Early surgical intervention is crucial to avert the high risk of associated morbidity and mortality. While conducting emergency laparotomy for intestinal obstruction, the rare type of primary IH as seen in our case should be kept in mind.
